# An Exemplary National COVID-19 Vaccination: Lessons from Bhutan

**DOI:** 10.3390/tropicalmed7070131

**Published:** 2022-07-11

**Authors:** Sangay Phuntsho, Tshokey Tshokey, Mongal Singh Gurung, Sonam Wangdi, Sonam Wangdi, Sonam Wangchuk

**Affiliations:** 1 Vaccine Preventable Diseases Program, Ministry of Health, Thimphu 11001, Bhutan; 2 Jigme Dorji Wangchuck National Referral Hospital, Thimphu 11001, Bhutan; doc_tshokey@yahoo.com; 3 Research Ethics Board of Health, Ministry of Health, Thimphu 11001, Bhutan; msgurung@health.gov.bt; 4 Policy and Planning Division, Ministry of Health, Thimphu 11001, Bhutan; sonamwangdi@health.gov.bt; 5 WHO Country Office, Thimphu 11001, Bhutan; wangdis@who.int; 6 Royal Centre for Disease Control, Ministry of Health, Thimphu 11001, Bhutan; swangchuk@health.gov.bt

**Keywords:** Bhutan, COVID-19, vaccination program, national campaign

## Abstract

Vaccination remains a key public health intervention against the COVID-19 pandemic. However, vaccine distribution and coverage are variable between countries due to access and implementation issues. Vaccine inequity was evident with some countries having no access to the vaccines while others have initiated multiple booster doses. We share Bhutan’s approach to COVID-19 vaccination and lessons learned during the successful conduct of a nationwide vaccination program. As of 12 December 2021, 80.3% of the Bhutanese population have received at least one dose of COVID-19 vaccine and 77.0% have received at least two doses. Considering age groups, 97.2% of adults (18 years) have received at least one dose and 93.6% have received at least two doses. The first dose coverage for the adolescents 12–17 years was 99.7% and second dose coverage was 92.3% since some were not yet due for their second dose at the time of writing this report. The well-established existing national immunization program was especially useful in the implementation of the national COVID-19 vaccination program. The Bhutan Vaccine System, a digital platform for registration and monitoring of vaccination, was rapidly developed and extensively utilized during the campaign. The selfless leadership of the king, the government, and prior detailed planning with multi-sectoral collaboration and coordination, was the key in this exemplary vaccination program. Bhutan has successfully vaccinated children between 5–11 years with high coverage and no serious issues. Many adults have also received first and second booster doses, based on their risks and preferences.

## 1. Introduction

An outbreak of severe acute respiratory syndrome coronavirus 2 (SARS-CoV-2) pneumonia was first reported in Wuhan, China, in late 2019. By the end of 2021, the virus has spread to 222 countries, infecting at least 281.808 million individuals, and killing more than 5.411 million people globally [1]. Although multiple public health measures and other mitigation strategies have prevented the massive transmission of SARS-CoV-2, it is unlikely that the world will return to its pre-pandemic normalcy until safe and effective vaccines were rolled out with high global vaccination coverage [2]. The first COVID-19 vaccinations started in early December 2020 and at least 13 different COVID-19 vaccines across four platforms have been approved for Emergency Use Authorization (EUA) by the respective country’s regulatory authorities till then [3]. In addition, by the end of 2021, there were 195 COVID-19 vaccines under pre-clinical development and 126 under various stages of clinical development [4]. At the time of writing this article, 58.1% of the world’s population have received at least one dose of COVID-19 vaccine, of which only 8.4% were in low-income countries, and a total of 9.1 billion doses were administered [5]. However, the global figure indicates huge inequalities between countries, continents, and income groups, and vaccine doses have so far been distributed unevenly among the low-income countries particularly those across Africa [6]. Months after availability of vaccines, countries still faced difficulties in allocating vaccines equally and ethically [7]. While several countries were struggling to reach the 40% coverage target by the end of 2021, other countries have vaccinated well beyond this threshold and also initiated extensive booster doses [8,9].

Bhutan started planning for COVID-19 vaccination since July 2020 and developed deployment plans, conducted cold chain assessment, human resource mapping, electronic recording and reporting system for real-time vaccination coverage, and AEFI monitoring. Under the benevolent guidance of His Majesty the King and the oversight of the Government, Bhutan conducted two rounds of nationwide COVID-19 vaccination campaigns achieving high coverage. Booster doses have also been provided to the eligible population. This report shares Bhutan’s experiences on conducting the nationwide COVID-19 vaccination rollout.

## 2. Methods

This study was conducted by a group of officials from the Bhutan Ministry of Health (MoH) closely involved in the planning, procurement, training, vaccination, and monitoring of the country’s COVID-19 vaccination campaign. It involved the review and analysis of official plans, guidelines, standard operating procedures, field reports, facts and figures maintained by the national vaccine program, and the day-to-day records of events during the national vaccination campaign. Data are presented as frequencies and proportions.

Administrative approval was granted by the MoH Policy and Planning Division, and ethics approval was waived by the Research Ethics Board of Health. There is no utilization of identifiable individual information and informed consent was not required.

## 3. Results

### 3.1. Bhutan’s Health System

Bhutan is a small country with about 750,000 people living in twenty districts. Medical care, including medicines and vaccines, are provided free of cost by the government as mandated by the constitution [10] and there are no private hospitals. These free services are provided through a three-tier system; (i) Primary health centers, sub-posts, and outreach clinics (ORCs) at the primary level, (ii) district or general hospitals at the secondary level, and (iii) regional and national referral hospitals at the tertiary level. Traditional and allopathic medicine services are fully integrated and delivered under one roof. At the grassroots level, village health workers (VHWs) play a key role in taking the basic health services to the people. There are 51 hospitals including referral hospitals and 186 primary health centers.

### 3.2. Bhutan’s Existing Immunization Program

Bhutan started the Expanded Program on Immunization (EPI) in 1979 with six vaccines (BCG, DTP, TT, OPV, and measles). Subsequently, several new vaccines have been introduced and integrated into the routine immunization program considering the national disease burden, financial implications, and health infrastructure. Recently introduced vaccines include hepatitis B, rubella, Hemophilus influenzae B, human papillomavirus (HPV), inactivated polio virus, mumps, pneumococcal (PCV), and seasonal influenza vaccine. Currently, Bhutan has 13 different vaccines in the EPI program.

Bhutan achieved Universal Child Immunization (UCI) in 1991, sustained neonatal tetanus elimination since 1994, received polio free certification in 2014, measles elimination certification in 2017, and hepatitis B control certification in 2018. The country has a well-established immunization program and has sustained high routine immunization coverage above 90% for the last several years [11]. Bhutan has conducted several nationwide vaccination campaigns in the past, including National and sub-national Immunization Days (NIDs/SNIDS) in early 1990s, and recently for measles and rubella, HPV and flu vaccination campaigns. Further, continued mop-up campaigns for measles and polio are carried out on regular interval in view of national, regional, and global elimination and eradication goals. Taking cue from all these experiences, Bhutan did not foresee many challenges in rolling out COVID-19 vaccines.

### 3.3. Early Planning through Programmatic Approach

Bhutan started planning the nationwide vaccination long before COVID-19 vaccines were even approved. The plan was to rapidly mobilize, distribute, and complete the nationwide vaccination campaign within a short period of time. The National guideline for COVID-19 vaccination was adapted from the WHO guideline^12^ on COVID-19 vaccination programs and AEFI reporting and management. All health workers were trained in this guideline before the national campaign. Health workers nationwide were adequately trained on the COVID-19 vaccination, and Adverse Events Following Immunization (AEFI) management and reporting. Vaccine cold chain storage capacity readiness assessment for all available COVID-19 vaccines were conducted and accordingly additional procurements were made in preparation for nationwide COVID-19 vaccination rollout. A dedicated risk communication and media team was established at the MoH and the Prime Minister’s Office (PMO). These two teams carried out rigorous public advocacies and awareness including sensitization of the media, local government officials and religious leaders on vaccination. They were able to adequately address vaccine hesitancy and gained adequate public trust and confidence in the vaccine and vaccination services. Bhutan only used vaccines that were granted Emergency Use Authorization (EUA) by the World Health Organization (WHO) and subsequently by the country’s Drug Regulatory Authority (DRA). These included Covishield, AstraZeneca, Moderna, Pfizer, and Sinopharm vaccines. A majority of these vaccines were donated by India, USA, European Nations, China, and the global COVAX facility, and some were purchased by the government.

### 3.4. National COVID-19 Vaccine Deployment Plan (NVDP) and Allocation Framework

In June, 2020, the WHO, GAVI, and the Coalition for Epidemic Preparedness Innovations (CEPI) launched the COVAX Facility and Advance Market Commitment (AMC) with the goal of ensuring rapid equitable access to safe and effective vaccines to all countries, regardless of income level. Bhutan also joined the GAVI COVAX AMC facility. With the confidence accorded by this facility in getting access to COVID-19 vaccine supply, Bhutan started planning for the deployment under different situations. As per the guideline from WHO [12] and guidance from the National Technical Advisory Group (NITAG), Bhutan developed comprehensive a National Vaccine Deployment Plan (NVDP) and allocation framework prioritizing the high-risk groups based on risk assessment [13]. The risk-based criteria were developed to prioritize the specific population groups for the vaccination, which includes risk of acquiring infection, severe morbidity and mortality, socio-economic impact, and risk of transmitting to others. These criteria were applied to specific population groups through a scoring matrix and phase-wise vaccination was recommended in the context of shortage of vaccine supplies initially. Phase one included high-risk healthcare workers, active front line workers, elderly 60 years and above, and people with chronic medical conditions. Phase two included passive front line workers and students 12 years of age and above, and staff in the schools and institutions, and phase three was for children less than 12 years and pregnant women. Although the registration and vaccination of pregnant women was encouraged, the final decision to get vaccinated was left to the individual. Children were to be vaccinated whenever the authorization was granted to the specific age-groups. The rest of the population who were not included under any of the above criteria were to be vaccinated in the fourth phase. The plan was kept dynamic, and changes were made whenever necessary based on evolving vaccines accessibility and availability, which helped in the smooth roll out of the vaccination campaign. Unlike most countries, Bhutan got adequate vaccine supplies for the entire eligible population for both first and second dose and rolled them out within a week without having to implement the phased manner which was initially envisaged and planned.

### 3.5. Bhutan Vaccine System (BVS)

In preparation for the COVID-19 nationwide vaccination, the MoH developed the Bhutan Vaccine System (BVS) specifically for the COVID-19 vaccination management. The BVS (https://bvs.moh.gov.bt, accessed on 1 June 2022) is a web-based portal aimed at ensuring quality data collection for evidence-based planning and management of the COVID-19 vaccination program. Months before the vaccination campaign, the BVS was operationalized, and all the eligible individuals were encouraged to pre-register their details online. However, those who could not pre-register were provided registration on site on the day of the vaccination at the respective vaccination posts. Information thus collected through BVS was used for data driven vaccination campaign planning (particularly vaccine distribution and designation of vaccination posts) and real-time monitoring of vaccination coverage and AEFI monitoring during the campaign. The BVS feature included registration, pre-screening, vaccination planning and scheduling, dashboard with vital information, real-time AEFI data and vaccination report generation, vaccine stock inventory, and a feature to self-generate a vaccination certificate with a QR code. The registration in the system was purely voluntary in nature and basic demographic information, such as name, age, gender, identity card numbers, and addresses were collected. The BVS was a game changer for the COVID-19 vaccination program in the country.

### 3.6. Vaccination Posts and Floor Plan during COVID-19 Vaccination

**Figure 1:**
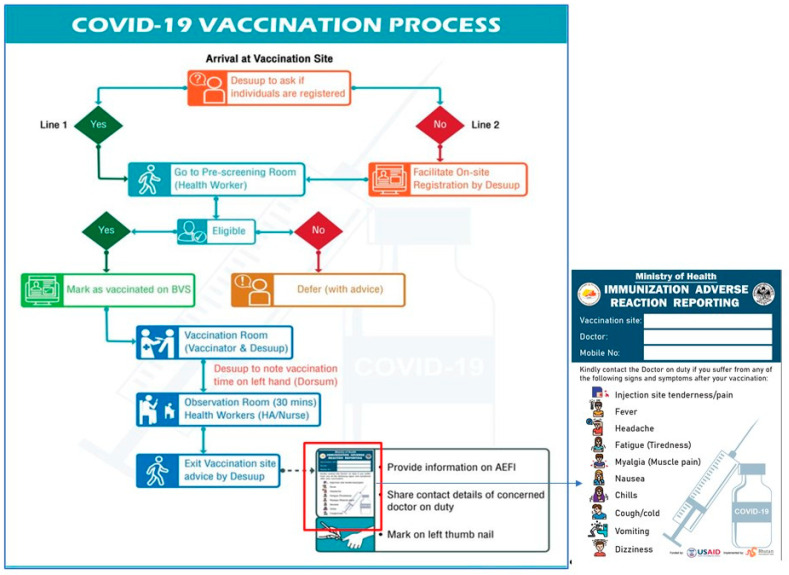
Algorithm for vaccination process.

Based on registration information of the eligible population in BVS, 1227 vaccination posts were set up in health facilities, schools, community centers, private dwellings, or temporary sheds across the country for the nationwide vaccination campaign. The schools that were identified as vaccination posts remained closed during the vaccinations. The MoH deployed more than 3600 health professionals to conduct pre-screening, vaccination, and AEFI observation and management during the campaign. In addition, about 4800 volunteers (Desuups: guardians of peace) were also deployed for crowd management, on-site registration, ensuring COVID-19 protocols were followed, and ushering the vaccinated individuals to the observation rooms and guiding their exit after fingernail marking to ensure people do not get double vaccination. This detailed pre-planning ensured smooth and seamless implementation of the campaign. Further, to ensure good vaccination coverage of all the eligible population within the allotted time frame of one-week, inter-district movements (except for emergency travels) were restricted. Most importantly, all these vaccination posts adhered to COVID-19 preventive and safety protocols with strict monitoring and supervision by the respective health workers, the national supervisors and officials from the Drug Regulatory Authority (DRA). The processes involved during the vaccination are depicted in Figure 1.

### 3.7. Nationwide COVID-19 Vaccine Distribution

**Figure 2:**
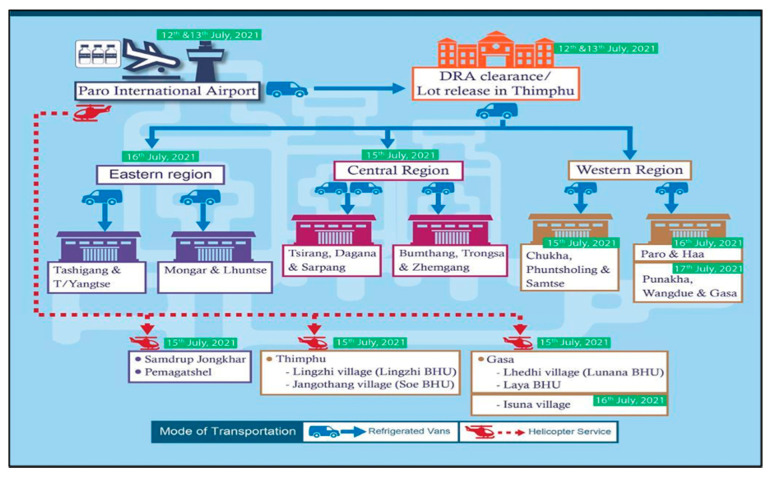
Vaccine transport and distribution plan.

The distribution of the vaccines commenced immediately upon arrival and lot release by the DRA. Vaccines were transported either by road in refrigerated vans, domestic flights and helicopter services, as per the distribution plan (Figure 2). The helicopter services were critical in shipping vaccines to the hard-to-reach places where there is no motor road access.

### 3.8. Launch of the Vaccination Campaign

Bhutan is a religious and spiritual country where astrology plays a significant role in the lives of the people. The government in consultation with the national astrologers chose an auspicious day (and time) in the Bhutanese calendar to launch the first nationwide vaccination campaign. The auspicious day fell on the 27 March 2021. Astrologers also recommended that the two auspicious people (the first dose vaccinator and the vaccinee) should be both females born in the monkey year and 30 years of age. Accordingly, on 27 March 2021, at 9.30 a.m., the campaign was launched in one of the vaccination posts in the capital city (Thimphu) followed by the launches in other districts.

### 3.9. AEFI Reporting and Management

The MoH developed AEFI monitoring and management of the BVS in line with the WHO guideline on COVID-19 vaccination program and AEFI reporting and management and trained all health workers on the guideline [14]. During the vaccination campaign, two or more vaccination posts had a mobile doctor deployed to guide, supervise, monitor and manage any AEFIs. As a precaution, all individuals were pre-screened for any risk factors before vaccination and were mandatorily kept under observation for at least 30 min after receiving the injection. For the convenience of the vaccinees, especially for those who reside away from health centers, prophylactic antipyretic (paracetamol) tablets were distributed after completing the 30-min observation. The NITAG and Regional Immunization Technical Advisory Group (RITAG) members were trained on COVID-19 vaccines AEFI management and stationed in the hotline centers during the campaign to monitor and provide technical guidance in addition to reviewing all the reported AEFI cases in BVS on a daily basis.

### 3.10. Target Population and Data Analysis

The target population was calculated based on the 2021 resident population of Bhutan which was estimated by the Civil Registration and Vital Statistics System (CRVS) of Bhutan and National Statistics Bureau (NSB). The population estimate included foreigners currently residing in the country and Bhutanese citizens who were out of the country. The vaccination data, including socio-demographic characteristics (age, sex, occupation, place of residence) and self-reported information on existing health conditions, allergies, and disability(s), were collected through BVS during the registration. AEFIs were collected during and/or after the vaccination. The data collected through BVS were exported and cleaned using EpiData software for missing values and outliers. The analysis utilized the cleaned data set after removing the duplicate entries and incorporating missing information which were re-validated through phone calls.

### 3.11. Vaccination Coverage

**Figure 3:**
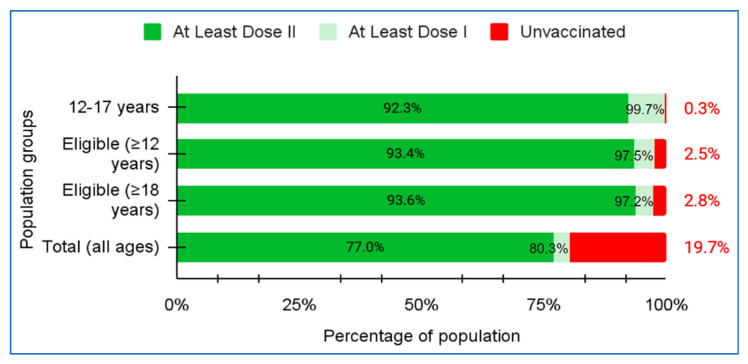
COVID-19 vaccination coverage for the first and second doses.

The vaccination coverage for different age cohorts for the first and second doses is shown in Figure 3. The overall population coverage was 80.3% and 77.0% for the first and second dose, respectively. In the adult population (18 years and above) the first dose coverage was 97.2% and second dose 93.6%. In the adolescents between 12 and 17 years, the first dose coverage was 99.7% and second dose 92.3%, respectively. As per the global vaccination status in December 2021, Bhutan stood at 14th highest share of fully vaccinated for COVID-19 vaccination coverage among the countries in the world [5].

### 3.12. AEFIs Reported during the Vaccination Campaign

AEFI cases were reported on a real-time basis through the BVS and managed immediately. The causality assessment was conducted by the national experts for all the serious AEFIs reported using the WHO causality assessment tool. The summary of number of minor and serious AEFIs reported against each vaccine type is shown in Table 1 below.

### 3.13. Vaccine Wastage Rate

Vaccine wastage is a huge concern due to the global shortage of COVID-19 vaccines and the cost implications. As per the WHO, vaccine wastage is calculated as:

- Vaccine usage rate = Number of doses administered/Number of doses issued × 100, and
- Vaccine wastage rate = 100 − vaccine usage rate

The overall wastage rate was 9.8% *(n* = 51,233 doses), with the wastage rate of 1.4% (*n* = 7382 doses) during the first dose vaccination campaign and 8.4% (*n* = 43851 doses) during the second vaccination campaign. The main reasons for the wastages were due to the short vaccine expiry date (85.1%), opened vials discarded after vaccination sessions (13.7%), broken vials (0.12%) and others (1.12%). Vaccine wastage rate was comparatively lower than the WHO acceptable wastage rate of 15% for multi dose vials containing 10 or more doses per vial [15].

### 3.14. Vaccine Hesitancy in Bhutan

As countries initiated vaccinations, vaccine hesitancy was a big issue. Reports on the COVID-19 vaccines being produced within short span of time, fake news, and widespread circulation of untrue information on social media most likely worsened vaccine hesitancy. Frank anti-vax is not known in Bhutan. The timely and effective risk communication and continued awareness program by the MoH and the PMO through various platforms (local government, monastic institutions, media houses, NGOs, social media influencers, etc.) were critical in reducing vaccine hesitancy and maximizing the vaccine uptake. A vaccine hesitancy online survey conducted prior to vaccination in the country in mid 2020 (*n* = 7476), revealed about 58% of the respondents would definitely take the vaccine and 27% would probably take it if it were made available. About 4% said they would not take the vaccine and 11% were undecided. However, when the actual vaccine arrived and the nationwide campaign started, many people chose to get vaccinated. Although the survey showed a significant number of unwilling or undecided people, during the campaign, most people took the vaccines, perhaps due to wide availability of the vaccine at the doorstep and increasing local and global confidences in the vaccines by the time vaccines were rolled out in the country.

### 3.15. Estimated Vaccination Program Cost

The estimated vaccine delivery costs (excluding vaccine costs) for the first and second dose cost per fully vaccinated individual is summarized in Table 2. The combined overall program cost for the first and second dose was USD 0.995 million (*First dose: USD 0.661 million, Second dose: USD 0.334 million*). The high program cost for the first dose was due to capacity-building of the health workers, cost for the BVS development, and procurement of immunization supplies and equipment which were not required during the second dose. These estimated costs were generated from records maintained by the Vaccine Preventable Disease Program with the assistance of the Ministry’s Administration and Finance Division.

## 4. Discussion

The first and the subsequent nationwide COVID-19 vaccination campaigns carried out in Bhutan are hailed as an example to the international community. Of all the things that contributed to this exemplary program is the leadership of the King, Prime Minister’s office, and the health and foreign ministries, together with detailed prior planning with a multi-sectoral collaborative approach and support provided by all relevant stakeholders. Building on an existing strong foundation of a national immunization program and experiences in conducting the nationwide vaccination campaigns in the past has helped health workers, districts, and central planners to implement the nationwide campaign successfully. Further, the use of the BVS for planning and vaccine distribution, real-time vaccination progress tracking and monitoring, and AEFI reporting and management was instrumental. The timely and effective risk communication and continued awareness program by the MoH and the PMO through various platforms were critical in reducing vaccine hesitancy and maximizing the vaccination coverage. The needs-based update in the vaccine deployment plan according to the real-time status update has helped minimize unforeseen challenges and allowed for smooth roll out of the vaccination campaign.

Post-vaccination campaign reviews and Inter-Action Review (IAR) meetings with relevant stakeholders, including representatives from the field after the first dose, were essential in making necessary improvements for the subsequent vaccination programs. For instance, during the first dose of adult vaccination, it was learned that some individuals who were alcohol dependent had stopped drinking a few days back prior to their vaccination schedule date, and this led to reporting of some alcohol withdrawal seizures adverse events at vaccination posts or at home after vaccination. Such adverse events were not reported during the second dose campaign after advocacy was carried out. During the first dose campaign, there were four incidences of people who received double doses of the vaccine, and with re-enforcement of proper procedures this was reduced to two incidences during the second campaign. Further, the implementation and experiences gained during the COVID-19 vaccination campaigns has contributed immensely to strengthening the overall immunization system in the country. The real-time immunization data management through BVS and overall cold chain capacity in the country has been strengthened. Building on the COVID-19 vaccines initiatives and to embrace the digital solution, all future vaccines are to be incorporated into BVS. To start with, the seasonal influenza vaccination for 2021 was already integrated into the system and HPV is planned in 2022, and subsequently all other childhood vaccines are to be administered using BVS. One of the strategies that has impacted in achieving high coverage is conducting a catch-up vaccination program after the campaign as a last mile to reach the maximum number of people, and also home-based and quarantine facility-based vaccination services. However, this approach was found to be time consuming and resource intensive.

The COVID-19 pandemic, which is probably the most devastating one in the last 100 years after Spanish flu, mandated the speedy evaluation of the multiple approaches for competence to elicit protective immunity and safety to curtail unwanted immune-potentiation which plays an important role in the pathogenesis of this virus [16]. The current COVID-19 pandemic has urged the scientific community internationally to find answers in terms of therapeutics and vaccines to control SARS-CoV-2 [17]. The United Nations and the WHO launched a strategy to vaccinate 40% of the world’s population against COVID-19 by the end 2021 and 70% by mid 2022 [17]. The earlier goal to vaccinate 10% of every country’s population by the end of September 2021 has fallen short, with 56 countries, mainly in Africa and the Middle East, unable to meet the target [17]. The vaccine production from various manufacturing companies has reached 1.5 billion doses per month and it is not a supply problem anymore but it is an allocation problem [17]. On the contrary, Bhutan has successfully vaccinated 80.2% of the overall population with a first dose and 76.8% with a second dose. Many have received booster doses. Children between 5–11 years have also been vaccinated without any issues. The COVID-19 vaccines must be a global public good and COVID-19 vaccines should contribute significantly to the equitable protection and promotion of human well-being among all people of the world. Safe and effective vaccines are a game-changing tool, and fair and equitable access to every country is of utmost importance to protect the people. Critics have argued that the high vaccination coverage in Bhutan is possible due to its relatively smaller population (less than one million people). However, it is noteworthy that many smaller countries more economically advanced than Bhutan have not be able to achieve the same results.

## 5. Limitations

This study was conducted after the completion of the second dose vaccination campaign in the country, and the data including the coverage for the subsequent doses, as well as vaccination for the children, are not included in this work. 

## 6. Conclusions

Bhutan has achieved a high vaccination coverage for both doses and boosters within a short time. This was attributed to the great leadership and detailed early programmatic planning. Utilization and building on the strong existing vaccination systems can aid in rapid national vaccination campaigns in outbreaks and pandemics. Digitalization of the current EPI program through the adoption of the BVS will contribute to the success of routine vaccination program in Bhutan.

## Figures and Tables

**Table 1:** Adverse Events Following Immunization rate for the first and second dose against different vaccines.

Vaccine Brand	First Dose			Second Dose		
	People Vaccinated (n)	*AEFI*		People Vaccinated (n)	*AEFI*	
		Minor ^1^	Serious ^2^		Minor ^1^	Serious ^2^
Covishield	491,266	17	8	18,651	3	0
AstraZeneca	5090	6	0	28,395	4	4
Pfizer	71,257	2	0	21,234	2	0
Moderna	22,350	27	9	496,798	8	2
Total	589,977	15	7	565,091	7	2

^1^ Per 1000 vaccinated people; ^2^ Per 100,000 vaccinated people.

**Table 2:** Cost distribution by activity and cost per fully vaccinated, Bhutan, 2021.

Activity	Proportion (%)	
	First Dose	Second Dose
Startup costs		
Planning and preparation	11.5	11.5
Information advocacy and materials	7.1	1.1
Training and consultations	25.7	1.9
Subtotal startup cost	44.3	14.6
Implementation costs		
Personnel (travel per diem)	48.9	77.4
Cold chain and injection equipment	3.4	5.1
Vaccine transport	3.4	2.9
Subtotal Implementation cost	55.7	85.4
Cost per fully vaccinated (USD)	1.86 (*1st dose: USD 1.13, 2nd dose: USD 0.73*)	

## Data Availability

All data have been presented in the manuscript and raw data are available on request from the corresponding author.
